# Permanent ^125^I-seed prostate brachytherapy: early prostate specific antigen value as a predictor of PSA bounce occurrence

**DOI:** 10.1186/1748-717X-7-46

**Published:** 2012-03-26

**Authors:** Renaud Mazeron, Agathe Bajard, Xavier Montbarbon, Frédéric Gassa, Claude Malet, François Rocher, Sébastien Clippe, Gabriel Bringeon, Olivier Desmettre, Pascal Pommier

**Affiliations:** 1 Radiation Therapy Department, Centre Léon Bérard, 28, rue Laënnec, 69373 Lyon Cedex, France; 2 Brachytherapy Department, Institut de Cancérologie Gustave Roussy, 39, rue Camille Desmoulins, 94805 Villejuif Cedex, France; 3 Unité de Biostatistique et d'Évaluation des Thérapeutiques, Centre Léon Bérard, 28, rue Laënnec, 69373 Lyon Cedex, France

**Keywords:** Brachytherapy, 125 iodine permanent seeds, Prostate cancer, PSA, Bounce, Biochemical relapse

## Abstract

**Purpose:**

To evaluate predictive factors for PSA bounce after ^125^I permanent seed prostate brachytherapy and identify criteria that distinguish between benign bounces and biochemical relapses.

**Materials and methods:**

Men treated with exclusive permanent ^125^I seed brachytherapy from November 1999, with at least a 36 months follow-up were included. Bounce was defined as an increase ≥ 0.2 ng/ml above the nadir, followed by a spontaneous return to the nadir. Biochemical failure (BF) was defined using the criteria of the Phoenix conference: nadir +2 ng/ml.

**Results:**

198 men were included. After a median follow-up of 63.9 months, 21 patients experienced a BF, and 35.9% had at least one bounce which occurred after a median period of 17 months after implantation (4-50). Bounce amplitude was 0.6 ng/ml (0.2-5.1), and duration was 13.6 months (4.0-44.9). In 12.5%, bounce magnitude exceeded the threshold defining BF. Age at the time of treatment and high PSA level assessed at 6 weeks were significantly correlated with bounce but not with BF. Bounce patients had a higher BF free survival than the others (100% versus 92%, p = 0,007). In case of PSA increase, PSA doubling time and velocity were not significantly different between bounce and BF patients. Bounces occurred significantly earlier than relapses and than nadir + 0.2 ng/ml in BF patients (17 vs 27.8 months, p < 0.0001).

**Conclusion:**

High PSA value assessed 6 weeks after brachytherapy and young age were significantly associated to a higher risk of bounces but not to BF. Long delays between brachytherapy and PSA increase are more indicative of BF.

## Introduction

Permanent seed prostate brachytherapy has become a standard treatment for localized prostate cancer [1,2]. The follow up of patients treated with this technique is mainly based on PSA screening, with PSA levels decreasing slowly over years to a nadir. The value of the nadir has been correlated with patient clinical outcome, which has led some authors to propose a threshold for defining biochemical complete response (i.e. 0.5 ng/ml for patients with at least 6 years follow-up), but no consensus has been reached on this issue for a long time [3-5]. The American Society for Therapeutic Radiology and Oncology (ASTRO) consensus conference held in San Antonio in 1997 defined biochemical failure after exclusive adjuvant external beam radiotherapy (EBRT) of the prostate as three consecutive increases in PSA levels [6]. According to this definition, date of relapse was calculated retrospectively as the midway between the date of nadir and the date of the first ascent of PSA. However, many criticisms were made on this back dating system, and on the fact that this definition did not account for clinical progression or survival and was not applicable in case of associated hormonal therapy. In 2006, the RTOG-ASTRO Phoenix consensus conference defined biological failure as a rise of 2 ng/ml or more above the PSA nadir [7]. This definition was rapidly assessed and was proposed in 2006 for use in prostate brachytherapy [8]. However, the decrease of PSA after prostate brachytherapy may be disrupted by the occurrence of PSA bounces, defined as a transient increase of the PSA value with spontaneous correction, which are frequent after prostate brachytherapy and may mimic or be mistaken for recurrences when strictly applying biochemical failure definitions.

The main objectives of this study were to identify predictive factors of bounce occurrence and criteria distinguishing bounces from true biochemical relapses.

## Methods and material

Prostate brachytherapy with permanent iodine seeds was first used at the Centre Léon Bérard in November 1999 and so far more than 500 patients have been treated. In order to investigate PSA bounces and obtain a significant follow-up, our study population included all the men for prostate carcinoma classified in the low or intermediate group according to D'Amico et al. classification [9], with at least 36 months follow-up. Patients who received hormonal therapy or additional EBRT were excluded.

### Procedure

Two different brachytherapy techniques were consecutively used: a free seeds technique with use of the Mick applicator during the first 3 years, then the "FIRST" technique from Nucletron (Veenendall, The Netherlands) characterized by the use of a seed projector. During both periods, the prescribed dose was 160 Gy to the entire prostate (applying TG43 guidelines) and an intra-operative dosimetry was performed based on ultrasound delineation of the prostate and organs at risk. PSA testing were performed 6 weeks and 6 months after the brachytherapy, then every 6 months up to 5 years, and at least once a year thereafter. The frequency of PSA testing was usually increased to every 3 months for patients who experienced a PSA increase.

### Definitions

As suggested by the majority of authors, PSA bounce was defined as an increase of at least 0.2 ng/ml above the nadir, followed by a spontaneous decrease to or below pre-bounce level [10-22]. An isolated increase in PSA at the first time point, 6 weeks after implantation, was not considered as a bounce and PSA values corresponding to visits earlier than 3 months after brachytherapy were not taken into account in bounce screening. Alternative definitions of the bounce were applied for the description of the bounces (+0.1, +0.4 and +2 ng/ml), but the analysis were done using +0.2 ng/ml.

The duration of the bounce was defined as the time from the pre-bounce nadir to the first PSA level below this nadir. The magnitude of the bounce was defined as the difference between the nadir and the highest value of the peak. Time to onset was assessed by delay between brachytherapy and date corresponding to the first date where PSA increased by more than 0.2.

Biochemical relapse was defined according to Phoenix criteria (PSA nadir + 2 ng/ml). True biochemical relapse was defined as a PSA increase fulfilling Phoenix criteria but not bounce definition (spontaneous PSA decrease) or post-brachytherapy positive biopsy or start of a salvage treatment. In case of patients with a bounce and then a true biochemical relapse, data were treated in the analysis of bounce and relapse. In the case of patients having two or more bounces, only the first one was used for analysis.

PSA velocity (PSAV) and PSA doubling time (PSADT) were calculated with formula: PSAV = (PSAf - PSAn)/ΔT and PSADT = ln(2) ΔT/(ln(PSAf) - ln(PSAn)) where ΔT is the delay between PSAn (nadir) and PSAf (date of peak for bounce patients or date of true biochemical relapse). Date of true biochemical failure was defined as the delay between brachytherapy and the first date where PSA is higher than 2 ng/ml from the nadir, the date of post-brachytherapy positive biopsy or the date of start of a salvage therapy.

### Statistical analysis

True biochemical relapse-free survival delay was calculated from the date of the brachytherapy to the date of relapse. The probability of true biochemical relapse-free survival was calculated using the Kaplan-Meier method.

Clinical or dosimetric factors possibly predictive of bounces were assessed by logistic regression. Factors included in the univariate analysis were: patient age, tumour risk group according to D'Amico et al. classification (low vs. intermediate), pre-BT PSA, PSA value assessed 6 weeks after the brachytherapy, brachytherapy technique (free seeds vs. FIRST) and intra-operative dosimetric parameters (prostate volume, V144, D95, D90, total number of seeds and seeds density).

Potential predictive factors of bounces with a 0.1 significance level in univariate analysis were included in a multivariate logistic regression.

These factors were also tested to predict presence of true biochemical relapse with a logistic regression model.

Bounce patients were compared to patients with true biochemical relapse: PSAV, PSADT, nadir before rise and time to onset were tested with a wilcoxon test. In case of patients with bounce and relapse, they were considered only with bounce for this comparison.

All statistical analyses were done using SAS software v.9.1 for Microsoft Windows (SAS Institute, Cary, NC, USA).

## Results

198 men fulfilled the inclusion criteria and were included in the study. Their characteristics are detailed in Table 1. Median age was 67 years (49-80). 69 patients were treated with the free seeds technique and 129 with the FIRST technique. The majority of the patients were classified in the low risk group according to D'Amico et al. classification (71.2%), and the others in the intermediate group.

**Table 1:** Patients' characteristics

Age	Median (range)	67 (49-80)
IPSS status (before BT)	mean (SD)	3.6 (3.5)
	median (range)	3 (0-17)
	P25-P75	1-5

Technique	free seeds	69 (34.8%)
	FIRST	129 (65.2%)

Risk group (D'Amico)	Low risk	141 (71.2%)
	Intermediate risk	57 (28.8%)

Pre-BT PSA	median (range)	7.2 (0.4 - 18.4)
	P25-P75	5.5 - 8.7

Per-op. dosimetric data		

Prostate volume	median (range)	35 (14 - 61.9)
	P25-P75	27.8 - 40.4

D95%	median (range)	166 (105-220)
	P25-P75	154-180

D90%	median (range)	182 (131-235)
	P25-P75	171-195

V144Gy	median (range)	98.4 (82.7-100)
	P25-P75	96.8-99.5

# seeds	median (range)	74 (40-120)
	P25-P75	63-83

Seeds density (N/cc)	median (range)	2.1 (1.5-4.0)
	P25-P75	1.9-2.4

The median follow-up was 63.9 months (range 36 - 119.4), 88.4 months (range 36.3 - 95.6) for the former implantation technique and 52.9 months (range 36 - 81.9) for FIRST.

**Figure 1:**
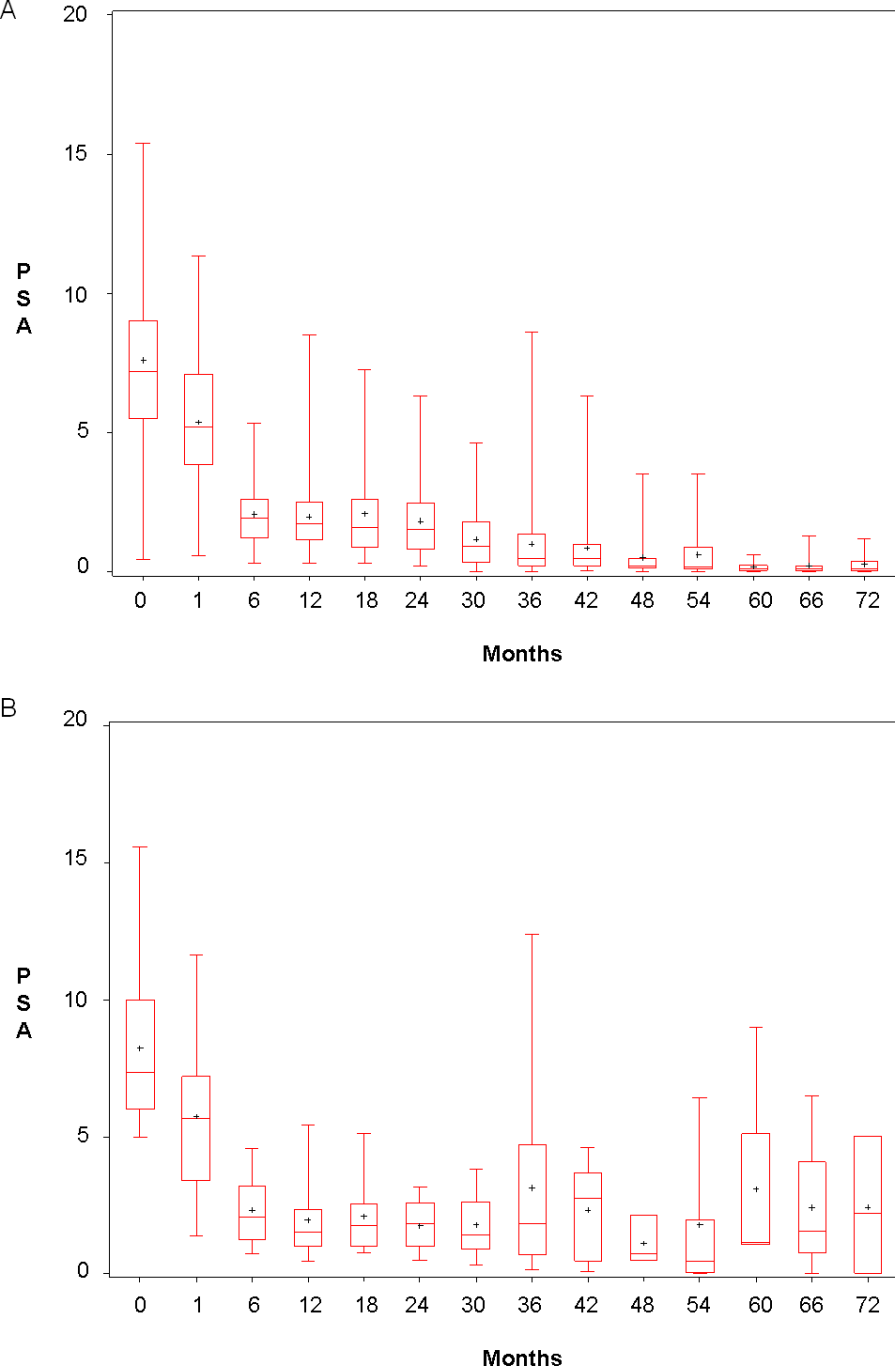
**A and B**. Distribution of PSA values per visit for patients who had bounce or relapsed. Figure 1A for bounce and B represents relapse.

A total of 2,219 PSA values were recorded, with a median number of 10.5 per patient (range: 3 - 24). Figure 1 shows distribution of PSA values at each visit.

At the first visit, scheduled 6 weeks after the brachytherapy (median 6.4 weeks, range 4.1 - 9.7), 20% of the patients had a PSA increase from its initial value (median increase: 0.83 ng/ml, range: 0.1 - 5.2). This value was not taken into account in bounce identification as it may have been altered by implantation and prostate edema.

At the time of analysis, 80.3% of the patients had achieved a nadir < 0.5 ng/ml.

### Bounces

Seventy one patients (35.9%) experienced at least one bounce defined by a PSA increase of at least 0.2 ng/ml followed by a spontaneous decrease to or below pre-bounce level. Ten patients experienced 2 bounces and one patient experienced 3 bounces. The respective proportion of bounces defined as a PSA increase with a threshold of 0.1, 0.4 and 2 ng/ml followed by a decrease to or below pre-bounce level were 48.5, 25.8 and 4.5% respectively. Characteristics of the bounces are presented in Table 2.

**Table 2:** PSA bounce characteristics according to bounce definitions

	Nadir + 0.2	Nadir + 0.1	Nadir + 0.4	Nadir + 2	Nadir + 15%	Nadir + 35%
**N (%)**	**71 (35.9%)**	**96 (48.5%)**	**51 (25.8%)**	**9 (4.5%)**	**103 (52.0%)**	**72 (36.4%)**

Delay						
Mean (SD)	18.5 (9.0)	18.7 (9.3)	18.6 (10.9)	16.5 (6.6)	21.5 (11.9)	24.6 (14.3)
Median (range)	17.0 (3.6-50.2)	16.7 (3.6-59.2)	17.4 (3.6-78.5)	17.9 (3.6-24.2)	17.9 (3.6-76.8)	21.4 (3.6-78.5)
P25-75	12.4-23.2	12.3-23.6	12.5-22.8	15.1-20.0	12.7-27.8	11.9-24.5

Magnitude						
Mean (SD)	1.0 (1.0)	0.7 (0.9)	1.2 (1.1)	3.2 (1.3)	0.7 (0.9)	0.9 (1.1)
Median (range)	0.6 (0.2-5.1)	0.4 (0.1-5.1)	0.8 (0.4-5.1)	2.5 (2.1-5.1)	0.4 (0.01-5.1)	0.6 (0.01-5.1)
P25-75	0.3-1.3	0.2-0.8	0.6-1.5	2.1-4.6	0.1-0.8	0.2-1.3

Duration						
Mean (SD)	16.5 (8.5)	15.2 (8.0)	17.7 (9.2)	24.8 (13.0)	16.6 (9.5)	18.8 (10.8)
Median (range)	13.6 (4.0-44.9)	12.9 (3.2-44.9)	15.9 (4.0-44.9)	29.3 (6.2-44.9)	13.6 (3.2-58.5)	16.8 (3.2-58.5)
P25-75	10.8-20.3	9.9-18.5	10.8-23.4	13.6-34.1	10.8-19.8	11.9-24.5

Delay for ascension						
Mean (SD)	7.9 (4.9	7.1 (4.7)	8.3 (5.2)	9.9 (5.9)	7.4 (4.9)	8.2 (5.6)
Median (range)	6.4 (1.3-23.0)	6.0 (0.4-23.1)	6.4 (1.5-23.0)	11.1 (3.0-19.0)	6.0 (0.4-23.1)	6.2 (0.4-23.1)
P25-75	4.4-11.0	3.7-8.3	4.4-11.1	4.9-11.8	3.7-10.6	3.8-11.7

Median time to onset was 17 months (3.6-50.2), and 56.3% of the bounces occurred between 12 to 24 months after the brachytherapy. After 30 months, bounces were rare (8.5%).

The median bounce duration was 13.6 months (range: 4.0-44.9), and 75% were limited to 20 months. 18.3% lasted over 2 years. The median increasing part duration of the bounces, which is the most agonizing, was 6.4 months (1.3-23), and was limited in 75% of the bounces to 11 months.

The median magnitude was 0.6 ng/ml (0.2-5.1). It was lower than 1 ng/ml in 72% of the cases, but higher than 2 ng/ml in 12.5% of the bounces (9 patients).

### Predictive factors for bounces

**Figure 2:**
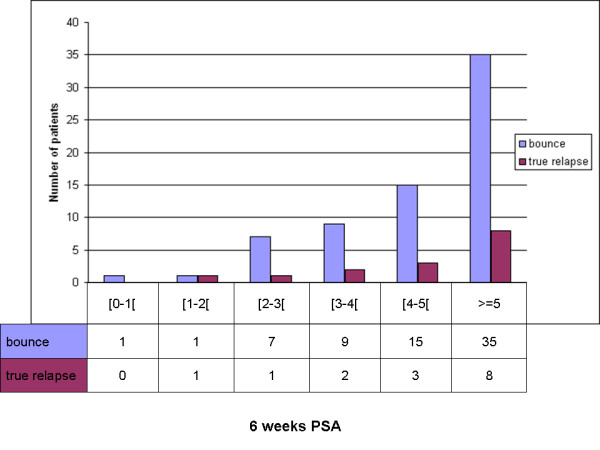
**6 week PSA value according to patients outcome: BF or bounce**.

In univariate analysis, younger age was significantly associated with a higher probability of a bounce occurrence (p = 0.003). There was also a trend for higher PSA value at 6 weeks (p = 0.053) (Table 3 and Figure 2). Dosimetric and clinical factors as well as initial PSA value and Gleason score were not associated with bounce.

**Table 3:** Predictive factors for bounce (univariate and multivariate analysis)

		Univariate analysis			Multivariate analysis		
**Variables**		**OR**	**CI 95%**	**p**	**OR**	**CI 95%**	**p**

Age		**0.936**	**0.895-0.978**	**0.0032**	**0.929**	**0.888-0.972**	**0.0016**

PSA at 6 weeks (wks)		**1.129**	**0.999-1.276**	**0.0526**	**1.166**	**1.025-1.327**	**0.0197**

Pre-BT PSA		1.044	0.941-1.157	0.4190	-	-	-

Risk group (D'Amico)	Int. risk vs low risk	1.180	0.625-2.229	0.6097	-	-	-

BT technique	FIRST vs Free seeds	0.666	0.364-1.217	0.1865	-	-	-

Increased PSA at 6 wks	Yes vs no	1.069	0.511-2.237	0.8584	-	-	-

Dosimetric data	D95 (D95)	1.009	0.994-1.024	0.2591	-	-	-

	D90 (D90)	1.007	0.990-1.023	0.4227	-	-	-
	V144 (V144)	1.075	0.954-1.211	0.2351	-	-	-
	Prostate volume	0.992	0.960-1.024	0.6166	-	-	-
	# seeds	0.997	0.975-1.019	0.7775	-	-	-
	Seed density	1.187	0.490-2.877	0.7037	-	-	-

In multivariate analysis, younger age (p = 0.0016, HR = 0.93, CI95%: 0.888-0.972) and higher PSA value at 6 weeks (p = 0.0197, HR = 1.17, CI95%: 1.025-1.327) were significantly associated with a higher probability of a bounce (Table 4). Patients with PSA bounce were significantly younger (median age 66 years (range: 51-79) vs. 69 years (49-80)) and had a higher PSA value at 6 weeks (median 5.2 ng/ml (0.6-11.3) vs. 4.3 (0.1-11.6)).

**Table 4:** Predictive factors for true recurrence (univariate analysis)

		Univariate analysis		
**Variables**		**OR**	**CI 95%**	**P**

Age		1.001	0.927-1.082	0.9734

PSA at 6 weeks (wks)		1.158	0.943-1.422	0.1626

Pre-BT PSA		1.081	0.907-1.287	0.3859

Risk group (D'Amico)	Int. risk vs low risk	1.260	0.411-3.863	0.6864

BT technique	FIRST vs Free seeds	0.586	0.203-1.690	0.3222

Increased PSA at 6 wks	Yes vs no	1.519	0.456-5.063	0.4964

Dosimetric data	D95 (D95)	0.989	0.962-1.017	0.4329
	D90 (D90)	0.985	0.956-1.015	0.3296
	V144 (V144)	0.965	0.805-1.156	0.6972
	Prostate volume	0.966	0.908-1.027	0.2681
	# seeds	0.966	0.926-1.009	0.1176
	Seed density	1.208	0.249-5.848	0.8145

### Biochemical failures

Strictly applying the RTOG-ASTRO Phoenix consensus definition, 21 patients (10.6%) were considered to have a biochemical relapse (nadir +2 ng/ml), resulting in a 4 year relapse free survival (RFS) of 92% (CI95% = 87-95). Six patients had salvage hormonal therapy (3 with positive biopsies), including 2 who did not meet the BF criteria (nadir +2 ng/ml).

In 9 cases, the PSA raise overcame the threshold of 2 ng/ml, but was followed by a spontaneous decrease to or below pre-bounce level fulfilling bounce definition. Thus, the "true recurrence rate" (true BF or salvage treatment) was 7.1% (21 BF + 2 salvage therapy - 9 bounces = 14 pts) and a 4 year RFS of 95% (CI95% = 91-97).

### Predictive factors for biochemical failures

"True recurrences" (definition above) occurred respectively in 5 pts belonging to the intermediate risk group (8.8%) and in 9 pts to the low risk group (7.1%) (p = 0.686).

In univariate analysis, no factor was found being predictive of true relapse. Results are shown in Table 4.

There was a better true biochemical relapse free survival in patients who experienced a bounce (true relapse free survival rate at 4 years: 100% vs 92%, logrank test: p = 0.0066).

Of the 71 patients who experienced a bounce, only one subsequently had a true biochemical relapse; he belonged to the intermediate prognosis group. Hence, none of the patients in the low risk prognosis group who had a bounce experienced a true biochemical relapse. Moreover, none of the patients who experienced a bounce > 2 ng/ml experienced a true biochemical recurrence.

### Biochemical failures vs. bounce

One patient experienced a bounce followed by a true biochemical relapse. He was considered as bounce patient here. Table 5 presents results of comparison between the 71 bounces and the 14 true relapses.

**Table 5:** Comparison between true biochemical relapse vs bounce

	Bounce	True relapse	p
**N**	**71**	**14**	

Delay			
Mean (SD)	18.5 (9.0)	51.0 (22.3)	
Median (range)	17.0 (3.6-50.2)	44.0 (15.6-89.6)	< .0001
P25-75	12.4-23.2	35.7-70.8	

PSA doubling time			
Mean (SD)	14.8 (13.3)	15.9 (14.3)	
Median (range)	11.5 (1.0-70.2)	12.0 (3.9-49.5)	0.995
P25-75	6.5-18.6	6.9-15.6	

PSA velocity			
Mean (SD)	0.2 (0.2)	0.2 (0.1)	
Median (range)	0.1 (0.01-0.9)	0.1 (0.03-0.6)	0.424
P25-75	0.1-0.2	0.1-0.2	

PSA nadir before increase			
Mean (SD)	1.6 (1.2)	1.2 (0.9)	
Median (range)	1.4 (0.02-6.6)	1.0 (0.04-3.0)	0.250
P25-75	0.8-2.3	0.5-1.9	

Delay where nadir +0.2			
Mean (SD)	18.5 (9.0)	31.5 (15.6)	
Median (range)	17.0 (3.6-50.2)	27.8 (7.8-61.7)	< .0001
P25-75	12.4-23.2	19.6-38.5	

Median PSADT was 11.5 months for the 71 patients who experienced a bounce (range: 1.0-70.2) and 12.0 months for the 14 patients with a true recurrence without bounce (range: 3.9-49.5). The median PSA velocity was estimated at 0.11 ng/ml/month for patients experiencing bounce (range: 0.01-0.85) and 0.12 ng/ml/month for patients with true relapse (range: 0.03-0.58). There was no difference between groups.

The median nadir before the rise was 1.38 ng/ml (range: 0.02-6.6) for bounce patients and 1.00 (range: 0.04-3.04) for true relapse patients (p = 0.25).

Bounces occurred significantly earlier than true BF (17 months vs. 44 months, p < .0001). Furthermore, the level of nadir + 0.2 ng/ml was reached significantly later in true BF cases than in bounce: 27.8 versus 17 months (p < 0.0001)

## Discussion

The long-known phenomenon of PSA bounce after prostate brachytherapy can lead to patient anxiety, unnecessary imaging exams or prostate biopsies, and even to inappropriate administration of salvage therapy. So far, the bounce mechanisms are not understood and their etiology unknown. It has been hypothetized to be linked to inflammation, radiation prostatitis or vascular fibrosis. Recently Kirilova et al. published prostate 3D MR spectroscopic assessments during PSA bounce in patients treated with permanent ^125^I seeds. They attempted to correlate the topography of the initial disease with the metabolic activity during the bounce and with the exact locations of local relapses in 24 patients [23]. They observed diffuse metabolic activity uncorrelated with residual malignancy or initial tumor mapping, suggesting that bounce could be related to inflammation.

### Bounce definition

PSA measurement has been shown to be a reliable method for monitoring but there is currently no consensual definition of a bounce. For most authors, bounces correspond to any variation beyond a given threshold above the PSA nadir, usually +0.2 ng/ml [10-22]. Alternative definitions have also been used: 0.1 or 0.4 ng/ml or an elevation compared to the previous nadir, presented as a ratio: 15% or 35% [15,24,25]. The definition should take into account physiologic fluctuations and laboratory assay variability. One standard deviation has been evaluated as 0.1 ng/ml. Depending on the definition applied bounce occurrence is highly variable and ranges from 2.5 to 88% (Table 6) [10-22,24-34]. However, three studies focusing on bounces and applying similar inclusion criteria (no additional pelvic EBRT or hormonal therapy) and similar bounce definition reported closer bounce rates, between 37 and 50% (details in Table 7) [14,21,34]. From those studies, the highest bounce rate was reported by Zwahlen et al. who included early PSA assessments in their analysis whereas we excluded the 6 week value from the bounce search as we consider that this value could be increased by the edema due to the seed implantation. This could be an explanation to their high bounce rate. The incidence and the time to occurrence of bounces may also be closely related to the frequency of PSA assessments after the brachytherapy, explaining some fluctuations. In that way, Caloglu et al. looking for a predictive factor of bounce occurrence showed that the number of PSA assessed per year was significantly correlated with bounce in multivariate analysis (1.8 vs 1.7, p = 0.014) [26].

**Table 6:** PSA bounce in literature

Authors	Pub	N	Median Follow-up (months)	Hormone Therapy (%)	EBRT (%)	Bounce Def	Rate (%)	Time to onset (months)	Magnitude (ng/ml)	Duration (months)	Predictive factors
Aaltomaa [10]	2009	444	81.6	yes (18)	Yes (4)	≥ 0.2 ng/ml	13	19.2	1.4	--	Age < 65, risk group, PSA nadir < 0.5

Bostancic et al. [11]	2007	164	65	yes (37.2)	no	≥ 0.2 ng/ml	26.9	18.9	0.5	8.7	Age,^125 ^I >^103 ^Pd *

Cavanagh et al. [12]	2000	591	55	no	yes	≥ 0.2 ng/ml	35.8	24.8	--	--	NC

Ciezki et al. [13]	2006	162	73	yes (38.2)	no	≥ 0.2 ng/ml	46.3	15	--	--	Age < 70 *

Caloglu [26]	2010	820	58	Yes (22.2)	no	≥ 0.2 ng/ml ≥ 0.4 ≥ 0.6 ≥ 0.8	30.1 19.6 12.8 9.5	17.4 16.3 16.2 15.7	--	--	Age, N of PSAs

Critz et al. [27]	2003	1,011	72	no	all	≥ 0.1 ng/ml	41	18	0.4	9	Age

Crook et al. [14]	2007	275	44	no	no	≥ 0.2 ng/ml	40	15.6	0.76	6.8	Age *

Das et al. [15]	2002	186	33	no	yes (25.8)	≥ 15%	62	26.4	0.6	12	--

Kanai et al. [17]	2009	86	32	no	no	≥ 0.4 ng/ml	33	15	0.6	--	Age < 67, D90 > 180 Gy

Kuban et al. [18]	2006	2,693	63	no	no	≥ 0.2 ng/ml	17	--	0.9	14	--

Hinnen et al. [16]	2011	975	78 ¤	Yes (19)	no	≥ 0.2 ng/ml	32.3	19.2	--	--	--

Makarewicz et al * [19]	2006	71	32	no	Yes (100)	≥ 0.2 ng/ml	31	13.5	0.28	--	Age, i PSA, V200

McGrath et al [28]*	2009	191	48	Yes (46%)	no	≥ 0.1 ng/ml ≥ 0.2 ng/ml ≥ 0.4 ng/ml ≥ 2 ng/ml	44 34 21 3	--	0.2 0.3 0.6 2.6	15 -- -- 25	--

Merrick et al. [20]	2003	218	46	no	yes (57.1)	≥ 0.2 ng/ml	23.9	16.3	0.9	16	Age, TNM, V150 PSA post brachy

Mitchell et al. [21]	2008	205	45	no	no	≥ 0.2 ng/ml	37	14.8	0.91	--	Age

Morita et al. [29]	2004	200	35	no	no	≥ 0.1 ng/ml	40	13	0.3	--	--

Patel et al. [22]	2004	295	38	yes (62.4)	no	≥ 0.2 ng/ml	28	19.4	0.5	--	Age < 65 *

Pickles et al. [30]	2006	449	48	yes (70)	no	all	84	13	--	--	--

Satoh et al. [24]	2008	388	--	no	no	≥ 0.1 ng/ml ≥ 0.4 ng/ml ≥ 35%	50.8 23.5 19.4	--	0.4	--	--

Stock et al. [25]	2003	373	48	no	no	≥ 0.1 ng/ml ≥ 0.4 ng/ml ≥ 35%	31 17 20	19.5 19.5 20.5	--	--	Age < 65, prostate volume > 35 cm^3 ^(bounce > 0.4)*

Thompson [31]	2010	1,006	54	Yes (65.7%)	no	+2 then nadir ≤ 0.5 ng/ml	2.5	20.6	--	--	Age

Toledano et al. [32,33]	2006	295	42	yes (42.4)	no	≥ 0.1 ng/ml ≥ 0.2 ng/ml ≥ 0.4 ng/ml	55 49 32	19	0.8	11.2	Age < 70, D90 > 200 Gy *

Zwahlen [34]	2010	194	60	no	no	≥ 0.2 ng/ml ≥ 0.4 ng/ml ≥ 15% ≥ 35%	50 34 11 9	14 14 16 15.5	05 0.8 1.9 2	12 13 18 21.5	Age

Mazeron (this study)	2012	198	64	no	no	≥ 0.1 ng/ml ≥ 0.2 ng/ml ≥ 0.4 ng/ml ≥ 15% ≥ 35%	48.5 35.9 25.8 52 36.4	17	0.6	14	Age, PSA 6 weeks

*: series including patients treated with HDR brachytherapy. --: non reported in publication. ¤: mean Abbreviations: Pub year = date of publication; N = number of patients; F-up, follow-up; Hormon, hormonal therapy. i PSA: pre treatment value of PSA. N of PSA: amount of PSA values recorded per patient

**Table 7:** Similar studies in detail

Authors	Year	N	Age	FU	% bounce	≥ +2 ng/ml	Delay	Amplitude	Nadir	duration	Bounce cause	BF & bounce	Alternative definitions
Crook et al. [14]	2007	275	64 (45-80)	44	40	15%	15.2 (3-29)	0.76 (0.21-11.79)	--	6.8 (3-50)	Age	Delay	--

Mitchell et al. [21]	2008	205	62 (43-75)	45	37	7.5%	14.9 (1.7-40.6)	0.91 (0.2-5.8)	1.4 (0.1-6.9)	11.3 (2.3-32.5)	Age	V PSA (using Phoenix def)	--

Zwahlen et al. [34]	2010	194	62 (47-75)	60	50	11.3%	14 (0-70)	0.5 (0.2-8.3)	0.1 (0-3.5)	12 (2-43)	Age	Delay	≥ 0.4 ng/ml: 34
													
													≥ 15%: 11
													
													≥ 35%: 9

Mazeron (this study)	2012	198	67 (49-80)	64	36	12.7%	17 (3.6-50.2)	0.6 (0.2-5.1)	1.4 (0.02-6.6)	13.6 (4-44.9	Age	6 week PSA assessment delay	≥ 0.1 ng/ml: 49

													≥ 0.4 ng/ml: 26
													
													≥ 15%: 52
													
													≥ 35%: 36
													

### Predictive factors for bounce occurrence

Age is the most commonly reported predictive factor for a bounce (Table 6). It seems that young age could be correlated to bounce occurrence, and different thresholds have reported, from 60 to 70 years old. Critz et al. showed that patients who were ≤ 60 years old have a two fold risk of bounce than the patients ≥ 71 years old (57% vs 26%, p < 0.0001) [27]. Similarly in a recent report Thompson et al. showed that 60% of the observed PSA bounces occurred in young patients (≤ 59 years old), whereas they account for only 22% of true BF [31]. Age may, therefore, also have influenced the differences in reported bounce rates. However, patients seemed to be similarly aged in the study by Crook and ours: 66 years old (50-80) versus 67 (49-80) [14]. In the series by Mitchell, patients were younger (median: 62.1, 43-75) and the bounce rate was slightly higher, 37% [14,21].

Prostate volume (> 35 ml) has been identified as predictive of bounce by Stock et al. (23 versus 11% at 5 years, p = 00.1), but this correlation has not been observed by the other authors [25]. The transition zone volume to total prostate volume ratio has been assessed in two studies. A low ratio was significantly associated with bounce in the study by Merrick et al. [20], but no correlation was reported by Crook et al. [14]

Das et al. tried to correlate PSA bounce to various events. They reported that 23% of the bounces may be subsequent to ejaculation, cycling, invasive exams, or radiation proctitis, [15] but those are not supported by scientific evidence.

Intra-operative and post-brachytherapy dosimetric factors have also been largely studied. Stock et al. found that D90 > 160 Gy was predictive of bounce (38% versus 24% at 5 years, p = 0.04) [25]. Toledano et al. reported similar results with a higher threshold, 200 Gy [32,33]. Recently, Kanai et al. found in a multivariate analysis that high D90 was correlated with bounce occurrence [17]. Some have speculated that high doses could lead to a greater likelihood of inflammatory and thus to PSA bounce. This dose/bounce correlation was also convenient to explain the correlation between bounce occurrence and biochemical control. Conversely, in Merrick et al.'s experience bounces are more likely associated with a low V150 (< 55%) [20]. In most studies, as in ours, authors failed to demonstrate a link between dosimetric factors and PSA bounce.

Pre-brachytherapy PSA value has not been correlated with the occurrence of a bounce, except in Makarewicz's HDR brachytherapy experience. In this report, the investigators showed that patients who experienced a bounce had greater pre treatment PSA value than the others (16.7 ng/ml vs 14.7 ng/ml, p = 0.045) [19].

Nevertheless, Merrick et al. have reported a correlation between a first high post treatment PSA value and the occurrence of a bounce (1.2 ng/ml vs. 0.7, p < 0.001) [35], but in that study, PSA was evaluated every 3-6 months and the first evaluation date seemed to be variable. In our series, PSA level was systematically assessed 6 weeks after the implantation, and was shown to be highly predictive of the occurrence of a bounce. Moreover, this parameter was not correlated with the occurrence of a true biochemical recurrence. Unfortunately, we did not identify any threshold.

### Dose rate/Isotope

Most of the studies were based on ^125 ^I permanent implantation. Merrick stated that the use of ^103 ^Pd lead to a half likelihood of bounce (17% versus 33%, p = 0.002) [35]. Those results were confirmed in a randomized trial comparing the use of ^125 ^I and ^103 ^Pd led by Bostancic et al. They have shown by multivariate analysis that ^125 ^I is significantly associated with a higher frequency of bounce in hormone-naive patients (45.7% with ^125 ^I vs. 14% with ^103 ^Pd) and in patients receiving neoadjuvant hormonal deprivation (respectively 28.1 and 20.7%) [11]. The dose rate can also be modulated in brachytherapy by using HDR. McGrath et al. reported similar rates between LDR permanent seed brachytherapy (34%, n = 191) and exclusive HDR brachytherapy (36%, n = 93) [28]. Similarly, Makarewicz et al. reported equivalent bounce rate while combining EBRT with HDR brachytherapy (31%, n = 31%) [19].

### Hormonal therapy

PSA bounce phenomenon and hormonal therapy is more confusing as the spike could be a consequence of the end of hormonal deprivation and of the testosterone recovery. For Patel and Toledano, ADT had no influence either on bounce rate or its magnitude [22,32]. Similarly, Ciezki et al., observed similar bounce rates between ADT treated patients (45%) and hormone naïve patients (48.4%, p = 0.67) [13]. Conversely, Pikles reported higher bounce rates in the ADT group (89% versus 71%, p = 0.001) [30].

### PSA bounce: a predictive factor for biochemical control?

It has been hypothesized that PSA bounce after brachytherapy could be predictive of biochemical control, whereas it is known to be correlated with biochemical failure after EBRT. In a series of 4,838 patients treated with EBRT, Horwitz et al. observed a bounce (defined as a ≥ 0.4 ng/ml PSA increase) in 20% of cases, and this bounce was independently correlated to biochemical failure [36]. These results have been confirmed by several other reports [37,38].

Patel et al. analyzed a series of 295 patients treated with brachytherapy (combined with hormonal therapy in 2/3 of the patients), with quite a short median follow up of 38 months. They observed that the BF-free survival assessed using the ASTRO consensus was 100% in the bounce group (28% of the population) vs. 92% in other patients (p = 0.018) [22]. In an other study by Ciezki et al. with longer follow-up (73 months), biochemical-free survival rates for patients who experienced or not a bounce were respectively 96% and 79% (p = 0.015) using the ASTRO definition and 100% and 92% (p = 0.004) using the Phoenix criteria [13]. Recently, Hinnen et al. published a large study including 975 patients and showed a strong link between bounce and outcomes. Ten years freedom from BF, disease free survival, and overall survival were respectively 90%, 99% and 88% in case of bounce against 70%, 93% and 82% for "no bounce" patients. They also reported only one cancer death in the "bounce group" (0.32%), compared with 40 (6.05%) in the no-bounce group [16]. Furthermore, Caloglu et al. tested several bounce definitions (≥ 0.2, ≥ 0.4, ≥ 0.6, ≥ 0.8) and found that the only definition for which there was a significant difference in BF free survival between bounce and no-bounce patients was +0.2 ng/ml (5-year BF control rates: 97.7% vs 97%, p = 0.0011) [26].

In our series, we observed only one biochemical relapse after a PSA bounce in one patient who belonged to the intermediate prognosis group. With 64 months follow-up, the occurrence of a bounce was therefore statistically correlated to biochemical disease-free survival in the subgroup of patients with "favorable prognosis" (p = 0.039).

### Differentiate benign bounces from genuine biochemical relapses

Distinguishing a benign PSA bounce from genuine biochemical recurrences is a major issue. On one hand it would reassure most patients with a PSA raise, and on the other hand, permit detection of true relapses in order to avoid expensive investigations such as 18-F choline PET/CT or invasive biopsies. To date, only follow up permits distinction of bounce from true BF. As illustrated in this series, 4 patients experienced an increase of the PSA over +2 ng/ml, and had begun a spontaneous decrease of their PSA at the time of analysis (of more than 1 ng/ml for 2 of them), but still could not be classified as bounces as the PSA did not return to the nadir.

Despite these limitations, Phoenix criteria have appeared to be more accurate than the ASTRO criteria to predict clinical outcomes in prostate cancer patients treated with either EBRT or BT [7].

Kuban et al. led a comparison of 12 different BF definitions on a large series of 2,693 men treated with permanent seed brachytherapy. They concluded that nadir +2 ng/mg provides the best sensitivity/specificity balance (70% and 89% respectively) [18]. Pickles et al. came to the same conclusion with a smaller cohort [30]. However, using this definition as a surrogate for relapses still leads to false positive results, especially because of the bounce phenomenon. Crook and Mitchell reported 15% and 7.5% false positives [14,21]. In our series, 9 cases (41% of the patients who experienced a nadir + > = 2 increase) would have been considered as biochemical failures by strictly applying the Phoenix criteria. For those patients, prostate biopsy cannot reliably distinguish between bounces and biochemical relapses during the first 3 years. Reed et al. reported 8 cases of patients who underwent biopsies as their PSA level increased to 2.6 and 8.4 ng/ml above the nadir, 9 to 25 months after the brachytherapy. Biopsies showed residual cancer, but PSA spontaneously decreased to previous level in all patients [39].

In order to take into account the lack of specificity of the Phoenix criteria, several definitions have been proposed. Patel et al. have simply suggested that a bounce should never exceed the pretreatment PSA level [22]. This parameter could be appropriate for Mitchell's series where only one bounce exceeded the pretreatment level, but it would have led to false positivity for recurrence in 7% of our bounce patients and 15% of those reported by Crook et al., and therefore does not seem reliable [14]. Thompson et al. applied an alternative PSA bounce definition: Phoenix definition (+2 ng/ml) followed by a spontaneous decrease to ≤ 0.5 ng/ml, threshold which had been previously used by some authors as usefull criterion. 44% of the BF were reclassified as bounces [31]. As described in most series, the large majority of the bounces occur during the first 2 years. Based on this observation, Ghilezian et al. recently proposed an alternative BF definition: nadir + 5 for the initial 24 months, and then nadir +2. This definition might be a superior predictor for biochemical failure in patients treated with brachytherapy, particularly if aged < 60 years [40]. In our series, applying such a definition would lead to only one patient misclassified as BF.

In an attempt to differentiate bounce from true biochemical relapse, several parameters have been tested. Mitchell et al. reported a series of 205 patients, and observed 79 bounces defined as an increase of ≥ 0.2 from the nadir followed by spontaneous decrease to the nadir value or under, and 6 Phoenix true biochemical relapses. They found that PSA velocity was 0.08 ng/ml/month for bounces versus 0.28 ng/ml/month for true Phoenix biochemical relapses (p = 0.0005). Using the former ASTRO criteria, they did not observe any significant differences. The authors failed to demonstrate a predictive threshold for PSADT [21]. PSA velocity and PSADT were not significant in our study, possibly because of the lenghthy time interval between PSA assessments at the time of PSA raise (6 months), and the limited number of patients experiencing a nadir +2 ng/ml increase.

Time to onset of PSA increase has been shown to be useful to distinguish bounce and relapse. Merrick et al. reported that 83% of the bounces occurred in the first 30 months following brachytherapy [20]. Ciezki et al. have reported that failures occurred after a median of 22.3 months, using Phoenix biochemical failure definition, whereas bounces occurred after a median of 15.1 months (p = 0.013) [13]. Similarly, Crook et al. have observed bounces at 15.2 months and failures at 30.9 months (p = 0.02) [14]. Our study confirmed the validity of this criterion (17 versus 27.8 months, p < 0.0001). We also observed that the whole misleading bounces, higher than 2 ng/ml (9 cases), occurred within the 24 first months of the follow-up (median 17.9 months), whereas "true" BF occurred from 15.6 months with a median delay of 44 months.

## Competing interests

The authors declare that they have no competing interests.

Presented at the annual ASTRO meeting held in San Diego (31/10 - 4/11/2010).

## Authors' contributions

PP and RM coordinated the study. RM: data collection. AB, RM: statistical analysis. RM, PP, AB: manuscript preparation. All authors read and approved the final manuscript.
